# Carotid-cavernous fistula initially presented with persistent daily headache with promptly neurological progression. Case report

**DOI:** 10.1186/1129-2377-14-S1-P158

**Published:** 2013-02-21

**Authors:** S Ljubisavljevic, M Spasic, D Stojanov

**Affiliations:** 1 Clinic for Neurology, Clinical centre of Nis, Serbia and Montenegro; 2 Center for Radiology, Clinical centre of Nis, Serbia and Montenegro

## Introduction

Carotid cavernous fistulas (CCF) are a dural arteriovenous fistulas which include pathological communications between the arterial system and the venous cavernous sinus situated at the wall of the cavernous sinus (CS). It can be demonstrated by wide range clinical presentations, like ophthalmic signs and symptoms (proptosis, chemosis, and visual loss), cranial nerve pareses, bleeding from the cranial structures and intracranial hemorrhage. We report a case of a patient which has diagnosed with CCF initially presented with persistent daily headache followed with promptly neurological progression. Case report: Twenty eight years old women has presented with 3 months history of daily left side headache, predominantly localized in left eye profound, without free after analgotherapy. Parallel with headache development she described sensation as a heart sound in pain area. Her mother died since some brain AV malformations. On examination, she had no ophthalmoplegia, proptosis and chemosis with normal neurological status. Auscultation under her left eye has found a whir. Digital subtraction angiography was performed and revealed a left side ophthalmic and facial venous dilatation with CS dilatation during all the time beginning from early arterial phase. As a result of panangiography detected a direct fast flow CCF, without presentation of left ICA intracranial parts with promptly retrograde venous swelling into the ophthalmic and facial venous and petrous sinus. During three days in neurological examination have occurred fully left side ophtalmoplegia with diplopia, left side ptosis and hypestesis in V1 area. The patient was scheduled for endovascular therapy (performed stent assisted coil embolization with complete occlusion of the fistula).

## Conclusion

In the small number of cases CCF can be presented by minimal symptoms such as persistent daily headache. This condition must be diagnosed and treated promptly since it has a high risk of clinical progression.
